# BRCA1, BRCA2 and their possible function in DNA damage response

**DOI:** 10.1038/sj.bjc.6690814

**Published:** 1999-12

**Authors:** Z Kote-Jarai, R A Eeles

**Affiliations:** Cancer Genetics Team, Institute of Cancer Research, 15 Cotswold Road, Sutton, Surrey SM2 5NG, UK


*British Journal of Cancer* (1999) **81**(7), 1099–1102
© 1999 Cancer Research Campaign
Article no. bjoc.1999.0814

Review

### BRCA1, BRCA2 and their possible function in DNA damage response

**Z Kote-Jarai and RA Eeles**

Cancer Genetics Team, Institute of Cancer Research, 15 Cotswold Road, Sutton, Surrey SM2 5NG, UK

The recognition of DNA damage and DNA repair responses are coupled to transcriptional events. This coupling evolved in mammalian cells in order to stimulate DNA repair processes, induce additional repair mechanisms, activate checkpoint functions or apoptosis. In order to repair, recover and survive, mammalian cells must recognize DNA damage and consequently they require the induction of specific genes and proteins. Some universal, and possibly some other tissue-specific, signal transduction pathways lead to the activation of key transcription factors which then regulate downstream molecular events in the damage response. The regulation of DNA damage repair at the transcriptional level could be a primary response and thus altered gene expression may offer the first detectable molecular events in the process of DNA damage control. BRCA1 and BRCA2 both have transcriptional activator functions and are part of a big nuclear protein complex, which may act as a molecular sensor and signal transducer. The role of the p53 gene in DNA damage response is well established and recently it has been reported that both BRCA1 and BRCA2 gene products actively interact with TP53. Also, there are several studies demonstrating altered damage response in cells mutated for BRCA1 or BRCA2. Clearly, there is a role or perhaps more than one role for the BRCA1 and BRCA2 genes in DNA damage response, but the real question is where to place their action in this very elaborate multistep pathway?

It is estimated that 10% of breast cancer patients develop the disease due to the presence of a breast cancer predisposition gene (Easton and Peto, 1990). Nearly half of these patients are thought to have a mutation in the breast cancer predisposition genes, BRCA1 or BRCA2. The recent isolation of BRCA1 (Miki et al, 1994) and BRCA2 (Wooster et al, 1995) stimulated intensive scientific interest. Although a large amount of information is now available, including nucleotide sequence, mutation spectrum, cellular localization and protein structure, the molecular pathway in which BRCA1 and BRCA2 is involved in response to DNA damage remains to be elucidated.

BRCA1 and BRCA2 are tumour suppressor genes since the majority of tumours arising in members of BRCA1- and BRCA2-linked families show loss of heterozygosity at the relevant loci with the retention of the mutant allele (Neuhausen et al, 1994; Collins et al, 1995). The products of both genes are large nuclear proteins; the BRCA1 gene encodes a protein of 1863 amino acids, the BRCA2 gene a protein of 3418 amino acids. The primary amino acid sequences reveal only little information regarding the function of these genes and, although there are some similarities between their genetic structure, there is no sequence homology between them. However, a number of observations indicate that BRCA1 and BRCA2 may function in a similar pathway. Their tissue distribution and gene expression pattern are similar, their expression level is cell cycle regulated (Rajan et al, 1996) and they both interact with RAD51 (Scully et al, 1997; Sharan et al, 1998) and TP53 (Marmorstein et al, 1998; Zhang et al, 1998).

RAD51 plays a key role in homologous recombination and DNA double-strand break (DSB) repair; thus this interaction suggests a role for BRCA1 and BRCA2 in the DNA damage response. The BRCA2 protein directly interacts with RAD51 through the evolutionarily conserved BRC domains (Wong et al, 1997). The interaction of the BRCA1 and RAD51 proteins has also been mapped to a specific region of BRCA1 exon 11 (Scully et al, 1997*a*), although it is not known whether this interaction is direct or mediated by other proteins. Furthermore, recently it has been shown that BRCA1 and BRCA2 form a complex in vivo, as they can be co-immunoprecipitated from cell extracts (Chen et al, 1998). Mapping the site for this interaction showed that the BRCA1 C terminal region is important and that RAD51 does not serve as a bridge between BRCA1 and BRCA2. In meiotic cells BRCA1, BRCA2 and RAD51 are all localized on unsynapsed axial elements, which suggests that this complex participates in signalling pathways and/or recombination. In mitotic cells they also co-localize in nuclear dots, and after DNA damage they relocate into the proliferating cell nuclear antigen (PCNA) replicating structures suggesting a joint action in damage response (Chen et al, 1998). However, it is important to note that these interactions are not stochiometric and there is a pool of ‘free’ RAD51 in the cells and, similarly, only about 5% of the BRCA1 and BRCA2 are in complex with each other. This also shows the possibility of multiple functions of these proteins.

Several studies have demonstrated a relationship between BRCA1 and BRCA2 gene function and normal growth control. For example, BRCA1 and BRCA2 are induced at the G1/S boundary in normal mammary epithelial cells and in breast cancer cell lines stimulated to proliferate (Gudas et al, 1996; Vaughn et al, 1997). BRCA1 message and protein was also shown to be induced by the mitogenic activity of hormones (Marks et al, 1997). The mouse homologues brca1 and brca2 appear to function in normal growth control and their very similar expression pattern suggests analogous regulatory pathway. A recent study showed that the expression pattern of brca1 and brca2 are closely correlated in most tissues with PCNA nuclear staining, which is a marker for proliferating cells in S phase. Exception was found in the testes where brca1 expression precedes brca2 expression (Blackshear et al, 1998), thus their regulatory pathways are not identical. This

*Received 1 June 1999*
*Accepted 8 June 1999*

*Correspondence to:* RA Eeles

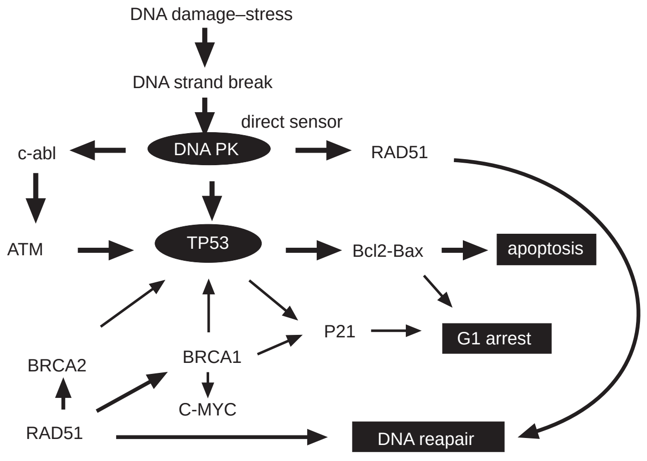

**Figure 1** TP53, a key responder to DNA damage, is interacting with BRCA1 and BRCA2 and they all take part in transcription regulation leading to the final cellular response of cell cycle arrest, DNA repair or apoptosis pattern during spermatogenesis also suggests that these genes can function beyond DNA replication, since brca1 and brca2 transcripts expressed for longer periods than the PCNA protein. Brca1, brca2 and rad51 nullizygous mice all show early embryonic lethality, associated with a proliferation deficit (Gowen et al, 1996; Ludwig et al, 1997; Suzuki et al, 1997) and it is very interesting to note that coincident homozygous p53 mutation can partially suppress this lethality (Ludwig et al, 1997).

The cells of BRCA1- and BRCA2-deficient tumours are often aneuploid (Marcus et al, 1996) and cells of brca2 mutant mice have aberrant chromosome structure coupled with inefficient DNA repair (Patel et al, 1998), which suggests that both genes are involved in the maintenance of genomic stability. Consistent with this hypothesis, brca2 nullizygous embryos exhibit X-ray hypersensitivity (Sharan et al, 1997). It has been shown that the human pancreatic adenocarcinoma cell line Capan-1, which has only one copy of the BRCA2 gene with a mutation producing a truncated protein eliminating the RAD51 interacting domain, has a striking deficiency in double-strand break repair (Abbott et al, 1998). These studies clearly demonstrate a role for BRCA1 and BRCA2 in DNA break repair and in the maintenance of genome integrity.

Looking into the protein structures of these gene products very little is revealed about the possible function. The BRCA1 protein has a RING finger in its N terminal globular region, which could be involved in DNA binding (Thakur et al, 1997). The tandem BRCT domain at the C terminal region (aa 1560–1863) has transcriptional activation function, as it transactivates gene expression when fused to a heterologous DNA binding domain (Monteiro et al, 1996). Furthermore, this BRCT motif is a relatively common feature of proteins involved in DNA repair or cell cycle checkpoint function (Bork et al, 1997; Calebaut et al, 1997). It has also been demonstrated that BRCA1 is physically associated with the RNA polymerase II complex (Scully et al, 1997) through RNA helicase A that interacts with the carboxy terminus of BRCA1 and links it to the complex (Anderson et al, 1998). Recent studies implicate that BRCA1 acts as a transcriptional co-activator and increases the TP53-dependent transcription from P21 and BAX promoters (Zhang et al, 1998). The interaction between BRCA1 and TP53 has been mapped to aa 224–500 of BRCA1 and the C terminal domain of TP53. These results would indicate that BRCA1 and TP53 may coordinately regulate gene expression; however, BRCA1 has also been found to transactivate the expression of P21, the major cyclin-dependent kinase inhibitor, in a TP53-independent manner (Somasundaram et al, 1997). These data suggest BRCA1 involvement in the inhibition of cell cycle progression into the S phase.

BRCA2 also has transcription activating function, which was localized to a highly conserved region at the amino acid terminus. Regions in exon 3 of BRCA2 show sequence homology with C-JUN and this exon has the potential to activate transcription in yeast (Milner et al, 1997). The BRCA2 protein has been shown to have histone acetyl-transferase (HAT) activity (Siddique et al, 1998), although this study was criticized, claiming that BRCA2 does not have intrinsic HAT activity but interacts with P/CAF1, a histone acetyltransferase (Fuks et al, 1998). Either way both studies suggest that BRCA2 participates in transcriptional regulation, as disrupting the nucleosomal structure through acetylation of certain histones is a possible mechanism for transcriptional activation.

Transcriptional response to DNA damage is well-documented after exposure of cells to different DNA-damaging agents. It has been shown that DNA-damaging agents trigger a transient induction of TP53 through post-translational mechanisms (DNA-PK, ATM) that inhibit ubiquitin-mediated degradation. The accumulation of TP53 in turn activates transcription from several genes, including P21, GADD45, MDM2, CYCLIN G, BAX. Post-translational modification, including phosphorylation by DNA-PK, may activate TP53 sequence-specific DNA binding or may affect TP53 activity through its interaction with other proteins. As BRCA1 has now been identified as a TP53-interacting protein (Zhang et al, 1998) and it also appears to regulate the expression of P21 in a TP53-dependent and -independent manner, there are strong reasons to propose an important role for BRCA1 in DNA damage processing.

Recently, a new protein, BAP1, has been found on the basis of its interaction with BRCA1 (Jensen et al, 1998). BAP1 is a ubiquitin carboxy-terminal hydrolase, suggesting that de-ubiquinating enzymes may play a role in BRCA1 function as well. BAP1 binds to the wild-type BRCA1 RING finger and enhances BRCA1-mediated growth inhibition of breast cancer cells. One possible model for BRCA1 function is that the BRCA1–BAP1 complex serves to target different substrates with its ubiquitin-mediated proteolysis. TP53 could easily be one of these target molecules and the RAD51/52 complex is also a candidate.

Surprisingly in a yeast two-hybrid system, c-Myc was also isolated as a BRCA1 binding protein and this c-Myc–BRCA1 interaction affects the cellular phenotypes caused by the synergistic actions of c-Myc and Ras. Mapping the interaction revealed two regions of BRCA1, aa175–303 and aa433–511. The small intervening region, which is rich in acidic amino acids, appears to account for some transcriptional activation function, although this activity is much lower than that of the C terminal domain. c-Myc is one of the early response genes activated in G1 phase and it activates the expression of a series of genes with important roles in cell cycling. Reduced c-Myc expression leads to a slower growth rate and delayed entry into S phase. It is possible that BRCA1 down-regulates Myc activity by preventing the Myc–Max heterodimer DNA binding or represses transcription through the action of BRCA1–Myc–Max complex. Either way this finding is another supporting evidence that BRCA1 is an important component of a transcription factor complex.

*© 1999 Cancer Research Campaign*

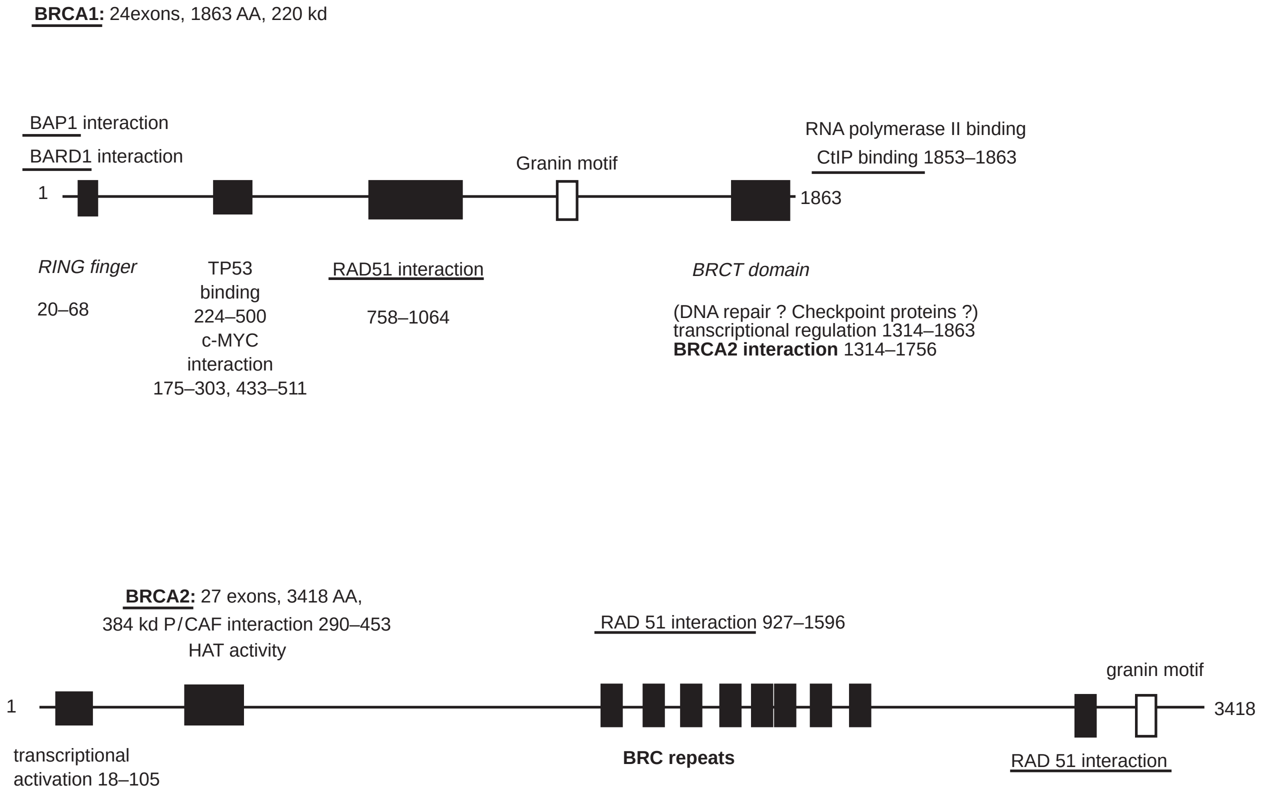

**Figure 2** Functional and structural elements of BRCA1 and BRCA2

In its unusually large exon 11, BRCA2 codes for eight tandem repeats, the BRC repeats, which are required for the RAD51 binding (Chen et al, 1998) and it was shown that this interaction is critical for cellular response to DNA damage. Very recently interaction has been demonstrated between BRCA2 and TP53 as well; however, no binding site was described and the interaction may be mediated by other proteins. It was shown that BRCA2 forms a complex with TP53 in vivo and this interaction has an important functional consequence. BRCA2 specifically inhibits p53 transcriptional activity (Marmorstein et al, 1998) and, most interestingly, the co-transfection with RAD51 and BRCA2 drove to almost complete inhibition of transcription activation by p53. This indicates that RAD51 enhances the BRCA2 inhibition of p53 transcriptional activity, proving that the interaction between BRCA2 and p53 has an important role in transcription regulation. It is possible that BRCA2 acts to limit the length of the p53-mediated cell cycle arrest. This is in concordance with a previous study that showed that the brca2/p53 double mutant embryo had less severe phenotype compared to the brca2 mutant one. In addition, brca2 mutant embryo cells exhibit increased p21 levels associated with a defect in proliferation. It is also a novelty that RAD51 is now linked to transcription regulation and perhaps BRCA2 serves as a bridge linking cell cycle control and DNA repair pathways.

An additional important feature of BRCA1 is that it becomes hyper-phosphorylated following DNA damage (Scully et al, 1997). In an earlier study of c-JUN induction by UV irradiation, it was found that UV exposure triggered a signalling cascade that increased phosphorylation of the c-jun protein resulting in enhanced transcription activation (DeVary et al, 1993). It is intriguing that BRCA1 may serve as a substrate for DNA-PK that upon DNA damage recognition phosphorylates BRCA1, just as it phosphorylates TP53 as one of the initial responses upon DNA damage. ATM can also play an important role in these molecular events as it was shown that ATM specifically phosphorylates TP53 after irradiation. Could BRCA1 also serve as a target for phosphorylation by ATM? Perhaps the BRCA1–Myc interaction is also affected by the BRCA1 phosphorylation state, and this way BRCA1 may act as a signal transducer from DNA damage to switch of cell cycle arrest versus apoptosis?

All the above data from recent studies strongly indicate that BRCA1 and BRCA2 have a primary role in DNA damage response by processing signals that arise after damage. Through cross-talks with other important elements of signal transduction pathways, BRCA1 and BRCA2 have an essential regulatory role in the cellular response to the damage resulting in cell cycle arrest, DNA repair or apoptosis. Some of the most recent studies show that both BRCA1 and BRCA2 may act upstream of the actual DNA repair and have important functions at the transcriptional level regulating genes taking part in the consequtive molecular events.

**Note added in proof** Since the preparation of this manuscript Harkin et al have shown that BRCA1 indeed has an important role in transcriptional regulation and GADD45, a DNA damage-responsive apoptosis gene, was found to respond with immediately elevated expression. This function of BRCA1 is proved to be p53 independent and further supports a role for BRCA1 in DNA damage response.

### REFERENCES

Abott DW, Freeman LF, Holt JT (1998) Double-strand break repair deficiency and radiation sensitivity in BRCA2 mutant cancer cell lines. JNCI **90**: 978–985

Anderson SF, Schlegel BP, Nakajima T, Wolpin ES and Parvin JD (1998) BRCA1 protein is linked to the RNA polymerase II holoenzyme complex via RNA helicase A. *Nat Genet* **19**: 254–257

Andres JL, Fan S, Turkel GJ, Wang JA, Twu NF, Yuan RQ, Lamszus K, Goldberg ID and Rosen EM (1998) Regulation of BRCA1 and BRCA2 expression in human breast cancer cells by DNA-damaging agents. *Oncogene* **16**: 2229–2241

Blackshear P, Goldsworthy SM, Foley JF, McAllister KA, Bennet ML, Collins K, Bunch DO, Brown P, Wiseman RW and Davis B (1998) Brca1 and Brca2 expression patterns in mitotic and meiotic cells of mice. *Oncogene* **16**: 61–68

Bork P, Hofman K, Bucher P, Neuwald AF, Altschul SF and Koonin EV (1997) A superfamily of conserved domains in DNA damage-responsive cell cycle checkpoint proteins. FASEB J **11**: 68–76

Callebaut I and Mornon JP (1997) From BRCA1 to RAP1: a widespread BRCT module closely associated with DNA repair. FEBS Lett **400**: 25–30

Chapman MS and Verma IM (1996) Transcriptional activation by BRCA1. *Nature* **382**: 678–679

Chen J, Silver DP, Walpita D, Cantor SB, Gazdar AF, Tomlinson G, Couch F, Weber BL, Ashley T, Livingston DM and Scully R (1998) Stable interaction between the products of the BRCA1 and BRCA2 tumour suppressor genes in mitotic and meiotic cells. *Mol Cell* **2**: 317–328

Chen P, Chen C, Chen Y, Xiao J, Sharp ZD and Lee W (1998) The BRC repeats in BRCA2 are critical for RAD51 binding and resistance to methyl methanesulfonate treatment. *Proc Natl Acad Sci USA* **95**: 5287–5292

Collins N, McManus R, Wooster R, Mangion J, Seal S, Lakhani SR et al (1995) Consistent loss of the wild type allele in breast cancers from a family linked to the BRCA2 gene on chromosome 13q12–13. Oncogene **10**: 1673–1675

Devary Y, Rowsette C, DiDonato JA and Karin M (1993) NF-kappa B activation by ultraviolet light not dependent on nuclear signal. Science **261**: 1442–45

Fuks F, Milner J and Kouzarides T (1998) BRCA2 associates with acetyltransferase activity when bound to P/CAF. *Oncogene* **17**: 2531–2534

Gowen LC, Johnson BL, Latour AM, Sulik KK and Koller BH (1996) Brca1 deficiency results in early embryonic lethality characterized by neuroepithelial abnormalities. *Nat Genet* **12**: 191–194

Gudas JM, Li T, Ngiyen H, Jensen D, Rauscher FJ and Cowan KH (1996) Cell cycle regulation of BRCA1 messenger RNA in human breast epithelial cells. Cell Growth Differ **7**: 717–723

Harkin PD, Bean JM, Miklos D, Song Y, Truong VB, Englert C et al (1999) Induction of GADD45 and JNK/SAPK-dependent apoptosis following inducible expression of BRCA1. Cell **97**: 575–586

Humphrey JS, Salim A, Erdos MR, Collins FS, Brody LC and Klausner RD (1997*a*) Human BRCA1 inhibits growth in yeast: potential use in diagnostic testing. *Proc Natl Acad Sci USA* **94**: 5820–5825

Jensen D, Proctor M, Marquis ST, Gardner HP, Ha SI, Chodosh LA, Ishov AM, Tommerup N et al (1998) BAP1: a novel ubiquitin hydrolase which binds to the BRCA1 RING finger and enhances BRCA1 mediated cell growth in suspension. *Oncogene* **16**: 1097–1112

Ludwig T, Chapman DL, Papaioanou VE and Efstratiadis A (1997) Targeted mutations of breast cancer susceptibility gene homologs in mice: lethal phenotypes of Brca1, Brca2, Brca1/Brca2, Brca1/p53, and Brca2/p53 nullizygous embryos. *Genes Dev.* **11**: 1226–1241

Marcus JN, Watson P, Page DL, Narod SL, Lenoir G, Tonin P and Linder-Stephenson L (1996) Hereditary breast cancer: pathobiology, prognosis, and BRCA1 and BRCA2 linkage. *Cancer* **77**: 697–709

Marks JR, Huper G, Vaughn JP, Davis PL, Norris J, McDonell DP, Wiseman RW, Futreal PA and Ingelhart JD (1997) BRCA1 expression is not directly responsive to estrogen. Oncogene **14**: 115–121

Marmorstein L, Ouchi T and Aaronson S (1998) The BRCA2 gene product functionally interacts with p53 and RAD51. *Proc Natl Acad Sci USA* **95**: 13869–13874

Mazoyer S, Puget N, Perrin-Vidoz L, Lynch HT, Serova-Sinilnikova OM and Lenoir GM (1998) A BRCA1 nonsense mutation causes exon skipping. *Am J Hum Genet* **62**: 713–715

Miki Y, Swensen J, Shattuck-Eidens D, Futreal A, Harshman K et al (1994) A strong candidate for the breast and ovarian cancer susceptibility gene BRCA1. *Science* **266**: 66–71

Milner J, Ponder BAJ, Hughes-Davies L, Seltman M and Kouzarides T (1997) Transcriptional activation functions of Brca2. *Nature* **386**: 772–773

Monteiro ANA, August A and Hanafusa H (1996) Evidence for a transcriptional activation function of BRCA1 C-terminal region. *Proc Natl Acad Sci USA* **93**: 13595–13599

Nelson WG and Kastan MB (1994) DNA strand breaks: the DNA template alterations that trigger p53-dependent DNA damage response pathways. *Mol Cell Biol* **14**: 1815–1823

Neuhausen SL and Marshall CJ (1994) Loss of heterozygosity in familial tumours from three BRCA1 linked kindreds. Cancer Res **54**: 6069–6072

Patel K, Yu VPC, Lee H, Corcoran A, Thistlethwaite FC, Evans MJ, Colledge WH, Friedman L, Ponder BA and Venkitaraman AR (1998) Involvement of Brca2 in DNA repair. *Molecular Cell* **1**: 347–357

Rajan JV, Wang M, Marquis ST and Chodos LA (1996) Brca2 is coordinately regulated with Brca1 during proliferation and differentiation in mammary epithelial cells. *Proc Natl Acad Sci USA* **93**: 13078–13083

Scully R, Anderson SF, Chao DM, Wei W, Ye L, Young RA, Livingston DM and Parvin JD (1997*a*) BRCA1 is a component of the RNA Polymerase II holoenzyme. *Proc Natl Acad Sci USA* **94**: 5605–5610

Scully R, Chen J, Plug A, Xiao Y, Weawer D, Feunteun J, Ashley T and Livingston DM (1997*b*) Association of BRCA1 and RAD51 in mitotic and meiotic cells. *Cell* **88**: 265–275

Scully R, Chen J, Ochst RL, Keegan K, Hoekstra M, Feunteun J and Livingston DM (1997*c*) Dynamic changes of BRCA1 subnuclear localisation and phosphorylation state are initiated by DNA damage. *Cell* **90**: 425–435

Sharan SK, Morimatsu M, Albreicht U, Lim D-S, Regel E, Dinh C, Sands A, Hasty P and Bradley A (1997) Embryonic lethality and radiation hypersensitivity mediated by Rad51 in mice lacking Brca2. *Nature* **386**: 804–810

Siddique H, Zou J, Rao VN and Reddy SP (1998) The BRCA2 is a histone acetyltransferase. *Oncogene* **16**: 2283–2285

Somasundaram K, Zhang H, Zeng Y, Houvras Y, Peng Y, Zhang H, Sheng Wu G, Licht D, Weber B and El-Diery WS (1997) Arrest of the cell cycle by the tumour-supressor BRCA1 requires the CDK-inhibitor p21. *Nature* **389**: 187–190

Suzuki A, de la Pompa JL, Hakem R, Elia A, Yoshida R, Mo R, Nishina H, Chuang T, Wakeham A, Itier A et al (1997) Brca2 is required for embryonic cellular proliferation in the mouse. *Genes Dev* **11**: 1242–1252

Thakur S, Zhang H, Peng Y, Le H, Carroll B, Ward T, Yao J, Farid LM, Couch FJ, Wilson RB and Weber BL (1997) Localization of BRCA1 and a splice variant identifies the nuclear localization signal. *Mol Cell Biol* **17**: 444–452

Vaughn JP, Davis PL, Jarboe MD, Huper G, Evans AC et al (1996) BRCA1 expression is induced before DNA synthesis in both normal and tumor derived breast cells. Cell Growth Differ **7**: 711–715

Wang Q, Zhang H, Kajino K and Greene M (1998) BRCA1 binds c-Myc and inhibits its transcriptional and transforming activity in cells. *Oncogene* **17**: 1939–1948

Wong AKC, Pero R, Ormonde PA, Tavtigian SV and Bartel PL (1997) RAD51 interacts with the evolutionary conserved BRC motifs in the human breast cancer susceptibility gene Brca2. *J Biol Chem* **272**: 31941–31944

Wooster R, Bignell G, Lancaster J, Swift S, Seal S, Mangion J, Collins N, Gregory S et al (1995) Identification of the breast cancer susceptibility gene BRCA2. *Nature* **378**: 789–791

Zhang H, Somasundaram K, Peng Y, Tian H, Zhang H, Bi D, Weber B and El-Diery WS (1998) BRCA1 physically associates with p53 and stimulates its transcriptional activity. *Oncogene* **16**: 1713–1721

Zheng H, Tombline G and Weber B (1998) BRCA1, BRCA2 and DNA damage Response: collision or collusion? *Cell* **92**: 433–463

 *© 1999 Cancer Research Campaign*
